# Automated measurement of cattle surface temperature and its correlation with rectal temperature

**DOI:** 10.1371/journal.pone.0175377

**Published:** 2017-04-20

**Authors:** HongXiang Kou, YiQiang Zhao, Kang Ren, XiaoLi Chen, YongQiang Lu, Dong Wang

**Affiliations:** 1 The Key Laboratory for Farm Animal Genetic and Utilization of Ministry of Agriculture of China, Institute of Animal Science, Chinese Academy of Agricultural Science, Beijing, China; 2 College of Animal Science and Technology, Jilin Agricultural University, Changchun, China; 3 State Key Laboratory for Agro-biotechnology, China Agricultural University, Beijing, China; 4 Animal Husbandry Station of Beijing, Beijing, China; Van Andel Institute, UNITED STATES

## Abstract

The body temperature of cattle varies regularly with both the reproductive cycle and disease status. Establishing an automatic method for monitoring body temperature may facilitate better management of reproduction and disease control in cattle. Here, we developed an Automatic Measurement System for Cattle’s Surface Temperature (AMSCST) to measure the temperature of metatarsus by attaching a special shell designed to fit the anatomy of cattle’s hind leg. Using AMSCST, the surface temperature (ST) on the metatarsus of the hind leg was successively measured during 24 hours a day with an interval of one hour in three tested seasons. Based on ST and rectal temperature (RT) detected by AMSCST and mercury thermometer, respectively, a linear mixed model was established, regarding both the time point and seasonal factors as the fixed effects. Unary linear correlation and Bland-Altman analysis results indicated that the temperatures measured by AMSCST were closely correlated to those measured by mercury thermometer (R^2^ = 0.998), suggesting that the AMSCST is an accurate and reliable way to detect cattle’s body temperature. Statistical analysis showed that the differences of STs among the three seasons, or among the different time points were significant (*P*<0.05), and the differences of RTs among the different time points were similarly significant (*P*<0.05). The prediction accuracy of the mixed model was verified by 10-fold cross validation. The average difference between measured RT and predicted RT was about 0.10 ± 0.10°C with the association coefficient of 0.644, indicating the feasibility of this model in measuring cattle body temperature. Therefore, an automated technology for accurately measuring cattle body temperature was accomplished by inventing an optimal device and establishing the AMSCST system.

## Introduction

As an important marker of both the health and physiological status of livestock, body temperature has been used to evaluate the regularity of oestrus and ovulation [1,2], pregnancy [3], parturition [4] and disease occurrence [5,6] in cows. However, manual measurement of body temperature is costly and time-consuming. It increases the potential risk of disease transmission and is impracticable to measure temperature over the course of a full diurnal cycle as well [7–11]. It is urgent, therefore, to establish a simple, precise, efficient and automated method for cattle body temperature detection.

**Fig 1:**
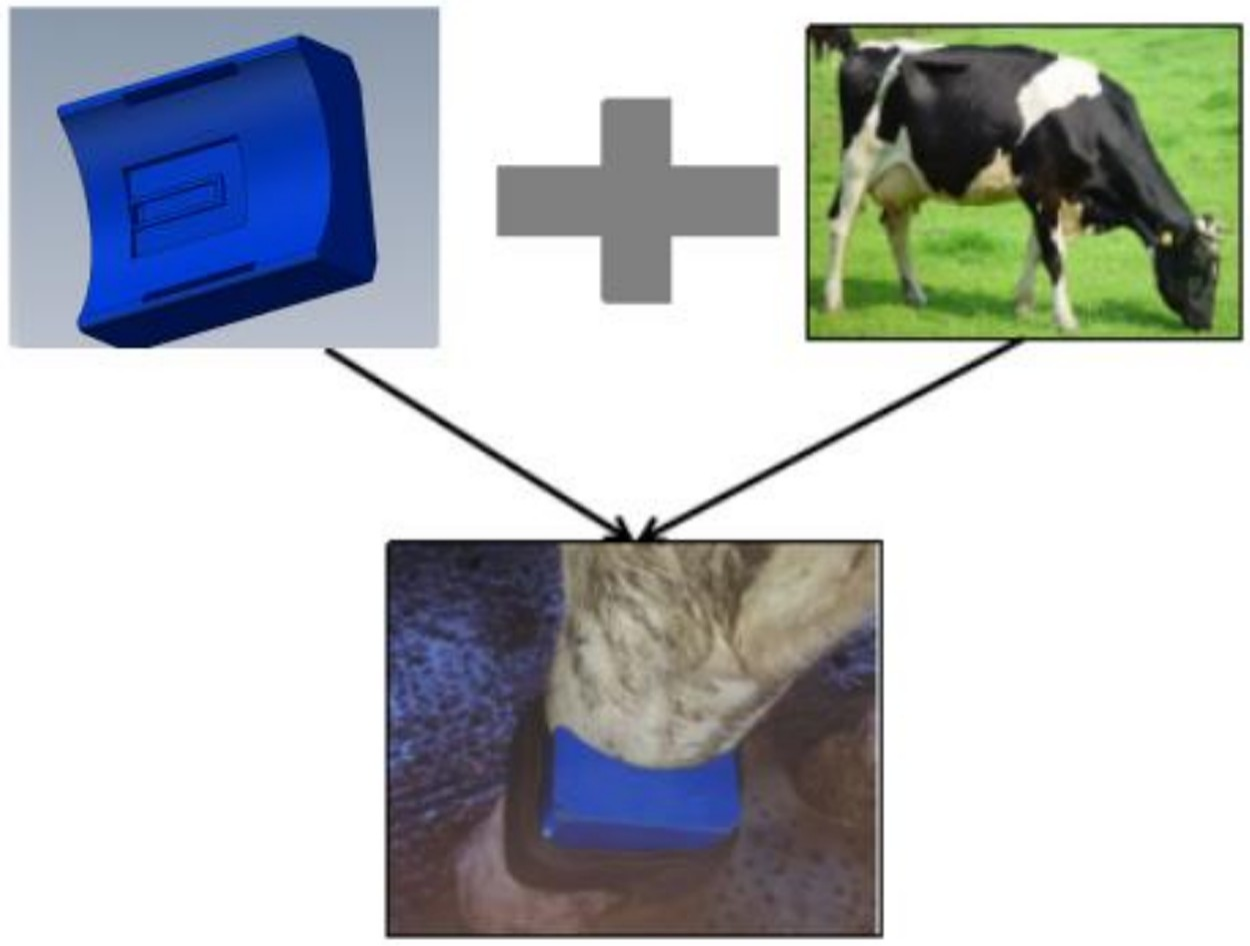
Installation diagram of the data detection device of AMSCST.

The rapid development of wireless telemetry technology laid a foundation for the automatic determination of body temperature. Early in 1974, Bligh proposed the use of wireless telemetry technology to measure animal body temperature [12]. This led to an expansion in the research of automated monitoring technology to detect dairy cow’s body temperature using infrared and implantable measurement devices. It is very difficult to accurately measure bovine body temperature in a non-contacted way due to its thick body hair. Vaginal implantation of wireless sensors made good progress in remote body temperature measurement of cows achieving an accuracy of 96.61% [13]. However, it has not yet been applied in the management of reproduction, because of lacking the supporting automated technology. Morais [14] and Miranda [15] reported the automatic detection of body temperature via implanting the temperature sensor into the cows’ vulvar muscles. Unfortunately, this approach cannot be used in dairy cows due to the vaginal damage. Automatic measurement of reticulo-rumen boluses temperature was attempted [5,16] but failed to return accurate body temperature due to the influence of water drinking and rumen fermentation. The measurement of dairy cow body temperature via automated detection technology based on infrared thermography (IRT) suggested that the eye, vulvar and the highest udder surface temperature (ST) are good proxies for rectal temperature (RT) [17,18]. Concerning the fact that IRT can be influenced by wind speed and direct sunlight exposure [19], it may be difficult to get accurate body temperature through analyzing IRT image [18,20]. The objective of this study was to establish a technology to automatically measure the body temperature of dairy cattle. We invented a specific device according to the anatomy of cattle’s hind leg, using which the ST on the metatarsus was successfully detected. The establishment of our Automatic Measurement System for Cattle’s Surface Temperature (AMSCST) based on thermistor sensor (Fig 1) provides a highly reliable and automatic cattle body temperature detection system.

## Materials and methods

In the present experiment, animal care and samples collection procedures were approved and conducted under established standard of the Institute of Animal Science, Chinese Academy of Agricultural Sciences, Beijing, China.

### Equipment

In this experiment, we used a commercially available mercury thermometer, veterinary mercury thermometers, as well as the AMSCST including seven sets of data detection devices, one for each of the seven animal, (animal physiological state detector in vitro: length 80 mm, height 74 mm, thickness 24 mm; patent number: CN201520869032.5) as depicted in Fig 1, a data collector, and one computer system.

The ST was measured using our AMSCST from the metatarsus of the hind leg with sparse hair. The temperature sensor is mounted inside the metatarsus where the muscle and blood vessels are rich. A shell shape was designed to fix the temperature sensor appropriate to the cattle anatomy. The temperature sensor was placed in close contact with the muscle via the shell and mounted on a concave nest positioned centrally on the side of the shell. This design minimizes the influence of environment factors such as wind, leading to more accurate temperature measurement.

### Testing and correcting the temperature measuring equipment

AMSCST was tested by placing 7 sets data detection devices of the AMSCST into the calorstat prior to the experiment to calibrate them. Firstly the temperature of the calorstat was increased from 30°C to 40°C using a steady increase rate of 1°C per 30 minutes, and then we automatically measured its temperature every 30 minutes using AMSCST. The temperature was also concurrently measured using a conventional mercury thermometer fixed to the inside of the glass door of the calorstat. This testing experiment was repeated three times. After the validation of its accuracy and reliability, AMSCST was applied to the cattle temperature detection.

### Animal feeding and management

Seven healthy Simmental cows, which were 60 to 90 days postpartum, multiparous and non-pregnant, were used as experiment animals. All cattle were housed in a free stall barn, fed five times daily (at 2:00, 6:00, 12:00, 18:00, 22:00) on an automatic total mixed rations (TMR) diet with drinking water freely available. Each cow was attached with one set of data detection device.

### Collection of the ST and RT

Both ST and RT were measured for periods during the winter (February 2nd to 8th), summer (June 2nd to 8th) and autumn (October 2nd to 8th) in 2015 in a cattle farm in Shijiazhuang city, Hebei province of China. Since weather in spring was considered similar to that in autumn, no measurement of ST and RT in spring in this study.

#### Carrying and adapting the data detection device

The data detection devices were attached to the metatarsus of testing cattle’s hind legs 3 days prior to the formal experiment to let the cattle get used to the device. ST was measured every 1 hour starting at 20:00 by data detection devices, and data were collected every four hours by the data collector from data detection devices.

#### Formal experiment

ST of cattle was continuously measured for 3 days using the AMSCST. Meanwhile RT was measured every 2 hours starting at 20:00 by a veterinary mercury thermometer. The testing cattle were held still for no less than 10 minutes prior to measuring the RT. The thermometer was removed 5 minutes after inserting into the rectum and the temperature was recorded. Each cow was continuously measured for 3 days and 12 RT data were collected per day.

### Statistical analysis

The t-test was used for the data obtained by the laboratory test section to find the significant difference. Relationship between the temperature data recorded by AMSCST and by mercury thermometer was evaluated using both Pearson correlation and linear regression. A Bland-Altman test [21] was used to test the consistency between the two measurements.

For each individual tested cow, 24 STs were obtained by AMSCST daily at an interval of 1 hour, while 12 RTs were collected per day at an interval of 2 hours by manual measurement using mercury thermometer. To match ST data set, the missing 12 RTs were arbitrarily generated by averaging two adjacent RTs since the difference of RTs between two adjacent time points was negligible. Totally, there were 24 STs and RTs data points for each cattle per day, and a total of 1512 paired STs and RTs data in all 3 tested seasons were obtained. We used the linear mixed model to estimate parameters for the fixed effects in regression. We also trained a linear mixed model to predict RT using ST adjusting for time and season. Only season or the season and time point are included as fixed effect in model 1 and model 2, respectively. In each model, we considered the variation between animals as well as covariation within animals for repeated measurements.

The models were established as follows:

$$\text{model }1:Y=\text{a}+b_{0}X_{0}+b_{s}X_{S}+b_{i}X_{i}+e;$$ (1)

$$\text{model }2:Y=\text{a}+b_{0}X_{0}+b_{s}X_{S}+b_{\text{t}}X_{t}+b_{i}X_{i}+e.$$ (2)

In the two models, Y represents RT, a represents intercept, X_0_ represents ST, b_0_ is the parameter vector of X_0_, X_s_ represents seasonal design matrix, b_s_ is the nonrandom parameter vector of X_s_, that is fixed effects; X_t_ represents time design matrix, b_t_ is the nonrandom parameter vector of X_t_, that is fixed effects. X_i_ represents the individual design matrix, b_i_ is the random parameter vector of X_i_, that are random effects; e is the vector of residual random errors. The outcome parameters are listed in the schedules. Determination coefficient (R^2^) is a measure of the reduction in variance when predictor variables have been added into the model. In SAS proc mixed, determination coefficient cannot be obtained directly, so we estimated residual from the full model(A) and model with intercept only (B), then used (B-A)/B to obtain the determination coefficient. RT was predicted based on ST by using the optional mixed liner model. The prediction accuracy was evaluated by comparing Pearson correlation coefficient, in a 10-fold cross validation approach [22]. For the training set, randomly picked 90% of the total 1512 data points were used to build the regression model in each run, and then the estimated parameters were used to predict RTs in the left 10% data points. The process was repeated 10 times. All analyses were carried out using SAS program.

## Results

### AMSCST test

**Fig 2:**
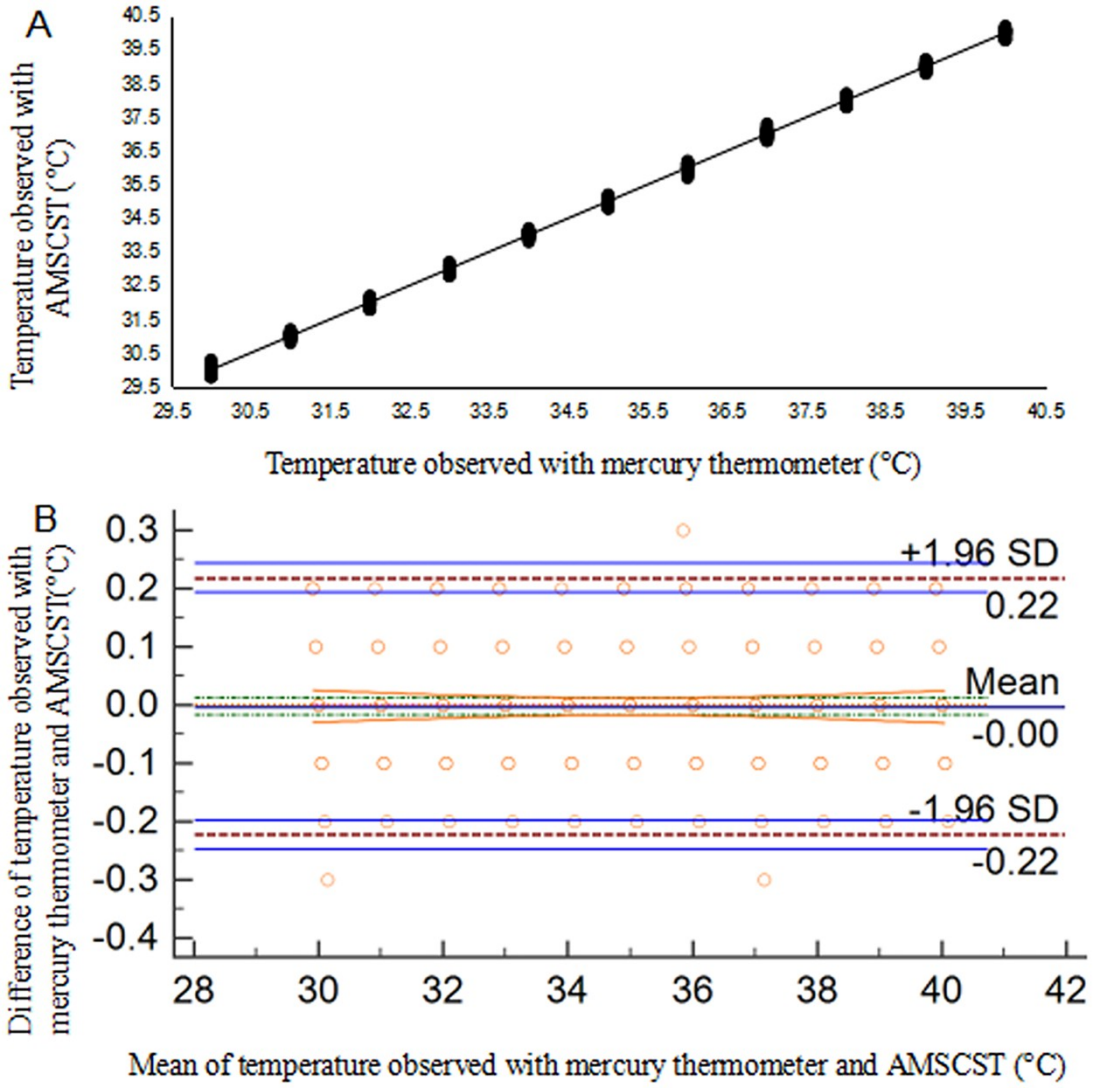
Consistency of temperature measured by AMSCST and mercury thermometer. A. The linear curve of the measured temperature of AMSCST and mercury thermometer; B. Bland and Altman plot showing agreement between measurements obtained by the mercury thermometer (as the reference) and AMSCST.

The pre-cattle laboratory test analysis demonstrated a close relationship between AMSCST and mercury thermometer measurement, with temperatures sourced from both devices synchronously increasing from 30°C to 40°C. The t-test analysis showed that there was no significant difference among AMSCST devices. The strong relationship between the temperatures detected by AMSCST and mercury thermometer (R^2^ = 0.998) was shown in Fig 2A, and the agreement between the temperature data measured by the seven sets of devices of the calorstat was shown in Fig 2B. The average difference between the temperature detected by different devices is 0.00 ± 0.07°C (95% CI: -0.22 to 0.22°C, Fig 2B). These results suggested the feasibility and reliability of AMTSCST which provides rather accurate and stable data in comparison with the conventional manual method.

### Analysis results of each factor

#### The ST and RT differences among days and seasons

There was no significant difference in RT within either days or seasons (*P*>0.05) (Table 1). This confirmed that the cow’s RT is nearly constant under the normal physiological conditions. Similarly, the ST difference among days was not significant (*P*>0.05) suggesting that AMSCST performance was fairly stable. However, ST varies by season with significant difference (P<0.05). The highest ST appeared in summer, while the lowest appeared in winter.

**Table 1:** The mean ST and RT in different days and different seasons (°C).

Measurement day	ST			RT		
	Winter	Summer	Autumn	Winter	Summer	Autumn
First day	34.31±1.97^A^	35.29±1.83^A^	34.28±1.61^A^	38.57±0.23^A^	38.44±0.17^A^	38.42±0.20^A^
Second day	34.16±1.80^A^	35.86±0.99^A^	34.43±1.52^A^	38.57±0.24^A^	38.52±0.16^A^	38.43±0.20^A^
Third day	34.22±1.92^A^	35.35±1.20^A^	34.29±1.53^A^	38.60±0.28^A^	38.47±0.13^A^	38.39±0.19^A^
Mean±SD	34.23±1.90^a^	35.50±1.43^c^	34.33±1.55^b^	38.58±0.25^d^	38.48±0.16^d^	38.41±0.20^d^

Note: A indicates there is no significant difference in the same column (*P* > 0.05);

a, b, c and d represent the differences in the same row, the same letter indicates there is no significant difference in the same row (*P* > 0.05), while the different letters indicate the group differs significantly from others in the same row (*P* < 0.05).

#### ST and RT differences across different daily time points

Table 2 shows that ST was significantly different from the measurement time point and largely consistent with the expected regular diurnal variation. The lowest ST appeared from 5:00 to 7:00, then gradually increased to peak value from 13:00 to 15:00. Subsequently, ST gradually declined again to another trough level. Similarly, RT was different significantly at different time points with similar pattern to that of ST although the diurnal variation of RT was marginal compared with ST.

**Table 2:** Diurnal mean ST and RT (°C).

Time	ST			RT		
	Winter	Summer	Autumn	Winter	Summer	Autumn
0:00	33.81±1.43^CD^	34.90±1.47^C^	33.79±1.20^BC^	38.53±0.17^C^	38.46±0.14^CD^	38.36±0.11^C^
4:00	33.73±2.04^E^	35.26±0.99^C^	33.32±0.84^CD^	38.34±0.22^D^	38.42±0.10^D^	38.31±0.08^C^
8:00	33.35±1.85^DE^	34.62±0.85^C^	32.90±0.96^D^	38.36±0.19^D^	38.26±0.09^E^	38.11±0.12^D^
12:00	35.98±1.03^A^	37.15±0.82^A^	35.86±0.78^A^	38.83±0.18^A^	38.56±0.11^B^	38.47±0.10^B^
16:00	35.07±1.51^AB^	36.31±1.46^B^	35.79±0.82^A^	38.71±0.19^B^	38.64±0.09^A^	38.75±0.14^A^
20:00	34.40±1.32^BC^	35.17±1.44^C^	34.10±0.90^B^	38.53±0.19^C^	38.51±0.09^BC^	38.49±0.09^B^

Note: A, B, C, D and E represent the differences in the same column. The same letter indicates there is no significant difference in the same column (*P* > 0.05), while different letters indicate the group differs significantly from others in the same column (*P* < 0.05).

### Regression analysis of ST to RT

#### Considering season as the only fixed effect

Linear mixed model 1 analysis was performed based on the ST and RT data, in which the seasonal factor was treated as the fixed effect as Eq 1.

The linear mixed model 1 was used to analyze the ST and RT data, and the estimated parameter values in this model were shown in S1 Table. The determination coefficient of the model is 0.399, which means that the model does not sufficiently fit.

#### Considering both season and time point as fixed effects

Since the determination coefficient of the previous model is 0.399, we then added time factor as fixed effect and obtained model 2 as Eq 2.

The estimated parameter values in model 2 are shown in S2 Table. The determination coefficient of model 2 was 0.562, thus providing a superior fit of the observed data.

#### Predicted RT

By employing the estimated parameters (in S2 Table) of season and time point in model 2, we obtained the predicted RT. The average difference between predicted RT and observed RT was 0.10°C. 90% (1360/1512) of the absolute differences were no larger than 0.2°C.

To evaluate the prediction accuracy of the above models, Pearson correlation coefficient between the predicted RT and measured RT was also calculated for the validation set upon 10-fold cross validation. The correlation coefficient of the model 2 is 0.644, which is higher than 0.569 from model 1.

## Discussion

Automated collection of body temperature data was pioneered using IRT to scan parts of human body. This process can assist in diagnosis a variety of diseases including breast cancer, local inflammation and vascular embolism [23–25]. However, the surface of the livestock such as cattle and horses is covered with thick hair, and hairless body parts such as eyes, udder and muzzle are not suitable for automated temperature detection because of their limited space for devices. Automated body temperature detection technology developed slowly, with few publications reporting the relationship between mastitis and udder ST measured by IRT [26,27]. Previoulsy, Metzner et al. measured udder ST using IRT, and reported its correlation with RT [18]. Because the udder ST can be markedly influenced by conditions such as mastitis, it is thus difficult to find an anatomical location for reliably monitoring the body temperature of dairy cows [27]. In our study, cattle’s ST was detected by measuring of the inside of the metatarsus of the cattle’s hind leg using our AMSCST. The design of shell shape of the AMSCST device greatly minimized the influence of environmental factors on body temperature, leading to more accurate and reliable measurement. Metzner [18] analyzed the relationship between RT and the udder ST’s maximum value and arithmetic mean of dairy cows using IRT. The results showed a better linear relationship between RT and the maximum value of the udder ST than RT and the arithmetic mean of the udder ST. The equation was RT = 5.68 + 0.874 × Tmax (Tmax represents the maximum value of the udder ST), and the determination coefficient was 0.646. However, the determination coefficient using RT and the arithmetic mean of the udder ST was smaller at 0.432. Coupled with the high cost and special equipment required by IRT, it is difficult to apply this approach in practice. Suthar et al. investigated the relationship between vaginal temperature (VT) and RT, and reported a determination coefficient between RT and VT of 0.846 for cows of 135 ± 56 days in milk and 0.884 for cows of 3 ± 1 days in milk respectively, indicating a strong relationship between RT and VT [28]. In fact the measuring duration in his study was only 80 ± 10 minutes with an interval of 1 minute, which was not long enough to reflect the variation of the body temperature. Another disadvantage is that the vagina is often affected by inflammation, and thus it is difficult to monitor the normal temperature of dairy cows. Furthermore, Suthar’s study only collected and analyzed the temperature for a limited period of time in the daylight hours, and didn’t consider the diurnal effect. The fitting curve may be difficult to reflect the regularity of cattle’s body temperature.

In this study, we compared two liner mixed models where the effect of time point was considered or not. An optimal model which included both season and time point, gave out a determination coefficient of 0.562. This value is 30% higher than the determination coefficient of 0.432 between RT and the average temperature of udder ST reported by Metzner. Furthermore, our detection system realized automation. Although the determination coefficient obtained by Suthar is higher, our method was easier to apply in practice since no stress response to animals when measuring ST compared with measuring VT. The predicted RT using this optimized model provides a good approximation to the actual observed RT, with the average difference of 0.10 ± 0.10°C, and 90% absolute difference is no bigger than 0.2°C. This indicates that the ST measured using our AMTSCST can be used as a reasonable estimation of cattle body temperature. Our invention may be extended to study other physiological phenomena displaying regularity such as estrus, pregnancy and parturition, and it can provide aid in disease diagnosis as well.

## Conclusions

This study described an automated technology for measuring cattle body temperature on the metatarsus of cattle’s hind leg. This method may play an important role in the future study for heat detection, pregnancy and disease diagnosis.

## Supporting information

S1 Table Considering season as fixed effects only. (DOC)

S2 Table Considering both season and time as fixed effects. (DOC)

S3 Table Supporting Information files of data. (XLS)
