# Supplementary material for: Automated measurement of cattle surface temperature and its correlation with rectal temperature
Source: PLoS One. 2017 Apr 20;12(4):e0175377. doi: 10.1371/journal.pone.0175377 (PMC5398510; doi:10.1371/journal.pone.0175377)
Supplement: S1 Table — (DOC) [file pone.0175377.s001.doc]

Schedule 1 Considering season as fixed effects only

| Effect | season | Estimate | Error | DF | t Value | Pr > |t| |
| --- | --- | --- | --- | --- | --- | --- |
| a |  | 35.5147 | 0.1064 | 6 | 333.81 | <.0001 |
| b_0_ |  | 0.08478 | 0.003054 | 1439 | 27.76 | <.0001 |
| b_s_ | 1 | 0.1607 | 0.01105 | 1439 | 14.54 | <.0001 |
|  | 2 | -0.02794 | 0.01156 | 1439 | -2.42 | 0.0158 |
|  | 3 | 0 | . | . | . | . |
