# Supplementary material for: Automated measurement of cattle surface temperature and its correlation with rectal temperature
Source: PLoS One. 2017 Apr 20;12(4):e0175377. doi: 10.1371/journal.pone.0175377 (PMC5398510; doi:10.1371/journal.pone.0175377)
Supplement: S2 Table — (DOC) [file pone.0175377.s002.doc]

Schedule 2 Considering both season and time as fixed effects

| Effect | season | time | Estimate | Error | DF | t Value | Pr > |t| |
| --- | --- | --- | --- | --- | --- | --- | --- |
| a |  |  | 37.1255 | 0.1236 | 6 | 300.48 |  |
| b_0_ |  |  | 0.03716 | 0.002599 | 1417 | 10.32 | <.0001 |
| b_s_ | 1 |  | 0.1557 | 0.009439 | 1417 | 16.49 | <.0001 |
|  | 2 |  | 0.02555 | 0.01026 | 1417 | 2.49 | 0.0129 |
|  | 3 |  | 0 | . | . | . | . |
| b_t_ |  | 1 | -0.01009 | 0.02612 | 1417 | -0.39 | 0.6994 |
|  |  | 2 | -0.06894 | 0.02612 | 1417 | -2.64 | 0.0084 |
|  |  | 3 | -0.05211 | 0.02164 | 1417 | -1.99 | 0.0464 |
|  |  | 4 | -0.09002 | 0.02615 | 1417 | -3.44 | 0.0006 |
|  |  | 5 | -0.08717 | 0.0262 | 1417 | -3.33 | 0.0009 |
|  |  | 6 | -0.1442 | 0.02626 | 1417 | -5.49 | <.0001 |
|  |  | 7 | -0.1283 | 0.02633 | 1417 | -4.87 | <.0001 |
|  |  | 8 | -0.1908 | 0.02619 | 1417 | -7.29 | <.0001 |
|  |  | 9 | -0.07616 | 0.02613 | 1417 | -2.91 | 0.0036 |
|  |  | 10 | -0.03006 | 0.02621 | 1417 | -1.15 | 0.2517 |
|  |  | 11 | 0.04516 | 0.02656 | 1417 | 1.7 | 0.0893 |
|  |  | 12 | 0.08145 | 0.02725 | 1417 | 2.99 | 0.0028 |
|  |  | 13 | 0.1618 | 0.0274 | 1417 | 5.9 | <.0001 |
|  |  | 14 | 0.1846 | 0.02773 | 1417 | 6.66 | <.0001 |
|  |  | 15 | 0.2104 | 0.02733 | 1417 | 7.7 | <.0001 |
|  |  | 16 | 0.1891 | 0.02669 | 1417 | 7.09 | <.0001 |
|  |  | 17 | 0.1811 | 0.02642 | 1417 | 6.86 | <.0001 |
|  |  | 18 | 0.1272 | 0.0262 | 1417 | 4.86 | <.0001 |
|  |  | 20 | 0.05839 | 0.02615 | 1417 | 2.23 | 0.0257 |
|  |  | 21 | 0.08796 | 0.02615 | 1417 | 3.36 | 0.0008 |
|  |  | 22 | 0.05663 | 0.02614 | 1417 | 2.17 | 0.0304 |
|  |  | 23 | 0.05352 | 0.02613 | 1417 | 2.05 | 0.0407 |
|  |  | 24 | 0 | . | . | . | . |
